# Protein kinase Cα regulates the nucleocytoplasmic shuttling of KRIT1

**DOI:** 10.1242/jcs.250217

**Published:** 2021-02-04

**Authors:** Elisa De Luca, Andrea Perrelli, Harsha Swamy, Mariapaola Nitti, Mario Passalacqua, Anna Lisa Furfaro, Anna Maria Salzano, Andrea Scaloni, Angela J. Glading, Saverio Francesco Retta

**Affiliations:** 1 Department of Clinical and Biological Sciences, University of Torino, 10043 Orbassano, Torino, Italy; 2 CCM Italia Research Network, National Coordination Center at the Department of Clinical and Biological Sciences, University of Torino, 10043 Orbassano, Torino, Italy; 3 Center for Biomolecular Nanotechnologies, Istituto Italiano di Tecnologia, 73010 Arnesano, Lecce, Italy; 4 Department of Pharmacology and Physiology, University of Rochester, Rochester, NY 14642, USA; 5 Department of Experimental Medicine, University of Genoa, 16132 Genova, Italy; 6 Proteomics & Mass Spectrometry Laboratory, ISPAAM, National Research Council, 80147 Napoli, Italy

**Keywords:** Cerebral cavernous malformation, KRIT1, PKC signaling, PKCα, PKCδ, Nucleocytoplasmic shuttling, Phorbol esters, Phosphoproteomics, Redox signaling

## Abstract

KRIT1 is a scaffolding protein that regulates multiple molecular mechanisms, including cell–cell and cell–matrix adhesion, and redox homeostasis and signaling. However, rather little is known about how KRIT1 is itself regulated. KRIT1 is found in both the cytoplasm and the nucleus, yet the upstream signaling proteins and mechanisms that regulate KRIT1 nucleocytoplasmic shuttling are not well understood. Here, we identify a key role for protein kinase C (PKC) in this process. In particular, we found that PKC activation promotes the redox-dependent cytoplasmic localization of KRIT1, whereas inhibition of PKC or treatment with the antioxidant N-acetylcysteine leads to KRIT1 nuclear accumulation. Moreover, we demonstrated that the N-terminal region of KRIT1 is crucial for the ability of PKC to regulate KRIT1 nucleocytoplasmic shuttling, and may be a target for PKC-dependent regulatory phosphorylation events. Finally, we found that silencing of PKCα, but not PKCδ, inhibits phorbol 12-myristate 13-acetate (PMA)-induced cytoplasmic enrichment of KRIT1, suggesting a major role for PKCα in regulating KRIT1 nucleocytoplasmic shuttling. Overall, our findings identify PKCα as a novel regulator of KRIT1 subcellular compartmentalization, thus shedding new light on the physiopathological functions of this protein.

## Abstract (precis)

**Summary:** A novel role for PKC signaling in triggering Ser/Thr phosphorylation and regulating nucleocytoplasmic shuttling of KRIT1, a major protein with pleiotropic functions associated with human diseases.

## INTRODUCTION

KRIT1 is a ubiquitous scaffolding protein with several emergent functions and a critical role in vascular morphogenesis and homeostasis. Loss-of-function mutations of the *KRIT1* gene have been clearly associated with the pathogenesis of cerebral cavernous malformation (CCM), a major vascular disorder affecting capillaries. CCM predominantly affects vessels in the central nervous system (CNS), and occurs in 0.3–0.5% of the general population (Batra et al., 2009; Cavalcanti et al., 2012). This disease is characterized by the formation of CCM lesions, also known as cavernous angiomas or cavernomas, consisting of clustered, abnormally enlarged and leaky capillary channels (caverns) lined by a thin endothelium and devoid of normal vessel structural components (Clatterbuck et al., 2001). CCM lesions can be single or multiple (up to several hundreds), and may remain asymptomatic or cause clinical symptoms of various type and severity, including recurrent headaches, focal neurological deficits, seizures and intracerebral hemorrhage (ICH) (Batra et al., 2009; Fontanella, 2015; Rigamonti, 2011).

Over the last decade there has been significant progress in understanding KRIT1 functions, providing useful insights into molecular mechanisms of CCM disease pathogenesis. Loss of KRIT1 has been shown to affect major cell structures and signaling mechanisms involved in the formation and stability of cell–cell and cell‒matrix junctions and the maintenance of endothelial and epithelial barriers, including the blood-brain barrier (Glading et al., 2007; Liu et al., 2013; Maddaluno et al., 2013; Stockton et al., 2010; Wei et al., 2020; Zawistowski et al., 2002; Zhang et al., 2001). Furthermore, accumulated evidence has clearly shown that the effects of KRIT1 loss-of-function on the stability of endothelial and epithelial barriers are due to an alteration of the complex machinery governing redox homeostasis and the cellular responses to oxidative stress and inflammation (Antognelli et al., 2018a,b; Choquet et al., 2016; Cianfruglia et al., 2019; Corr et al., 2012; Finetti et al., 2020; Gibson et al., 2015; Goitre et al., 2010, 2014, 2017; Marchi et al., 2015; Retta and Glading, 2016; Tang et al., 2017; Retta et al., 2020). Overall, this complexity has made a comprehensive understanding of KRIT1 function extremely challenging.

Useful insights into the molecular mechanisms underlying the biological roles of KRIT1 have been derived from the functional characterization of its structural motifs and domains, including the identification of specific interacting proteins. KRIT1 is a 736 amino acid protein that contains distinct protein–protein interaction domains, including a Nudix domain and three NPXY/F (Asn-Pro-x-Tyr/Phe) motifs within the N-terminal region, four central ankyrin repeats, and a C-terminal clover-shaped FERM domain (Fisher and Boggon, 2014; Zhang et al., 2015). This FERM domain is composed of three structurally unrelated subdomains (lobes F1, F2 and F3) featuring a ubiquitin-like fold, a four-helix bundle, and a phosphotyrosine binding (PTB)-like domain, respectively. Collectively, these multiple motifs, domains and subdomains form various binding sites for distinct interaction partners (Draheim et al., 2014; Fisher and Boggon, 2014). Known binding partners of KRIT1 include integrin cytoplasmic domain–associated protein 1α (ICAP1α, also known as ITGB1BP1) (Liu et al., 2013; Zawistowski et al., 2002; Zhang et al., 2001), cerebral cavernous malformation 2 (CCM2) (Fisher et al., 2015; Zawistowski et al., 2005; Zhang et al., 2007), sorting nexin 17 (SNX17) (Czubayko et al., 2006; Stiegler et al., 2014), the actin cytoskeleton-stabilizing protein Nd1-L (also known as IVNS1ABP; Guazzi et al., 2012), the membrane anchor protein heart of glass 1 (HEG1) (Gingras et al., 2012, 2013; Kleaveland et al., 2009), and the small GTPase Rap1 (Gingras et al., 2013; Li et al., 2012; Serebriiskii et al., 1997).

Scaffolding proteins such as KRIT1 are commonly regulated by localization in specific subcellular microdomains, which facilitates interactions with specific partner proteins and phospholipids. KRIT1 has been found in multiple cellular and subcellular compartments, including bound to microtubules, at cell boundaries and cell–cell junctions, and in the nucleus (Béraud-Dufour et al., 2007; Draheim et al., 2017; Francalanci et al., 2009; Glading et al., 2007; Glading and Ginsberg, 2010; Liu et al., 2011). Interactions between KRIT1 and corresponding binding partners, such as Rap1 or ICAP1α, appear to regulate KRIT1 trafficking between microtubules and the plasma membrane (Béraud-Dufour et al., 2007; Liu et al., 2011), or between the cytoplasm and the nucleus (Draheim et al., 2017; Francalanci et al., 2009; Su et al., 2020; Zawistowski et al., 2005; Zhang et al., 2007), respectively. In addition, the activity of most FERM domain-containing proteins (FDCPs), including the ezrin/radixin/moesin (ERM) family of proteins as well as merlin, talin, focal-adhesion kinases (FAKs) and Janus tyrosine kinases (JAKs), is known to be regulated by head-to-tail intramolecular autoinhibitory interactions involving the FERM domain, which in turn are regulated by phospholipid binding and phosphorylation (Fehon et al., 2010; Frame et al., 2010; Goksoy et al., 2008; Goult et al., 2009; Li et al., 2007; Lietha et al., 2007; Mishra et al., 2012; Pearson et al., 2000). We and others have also shown that KRIT1 can form intramolecular interactions between its N-terminal NPXY/F motifs and the C-terminal FERM domain (Béraud-Dufour et al., 2007; Francalanci et al., 2009). Specifically, we found that KRIT1 may adopt a closed conformation through a head-to-tail intramolecular interaction involving the third NPXY/F motif at the N-terminus [amino acids (aa) 250–254] and the PTB subdomain (aa 636–736) of the FERM domain at the C-terminus (aa residue numbers refer to isoform 1 of KRIT1; UniProt Q6S5J6-1) (Francalanci et al., 2009). Moreover, we demonstrated that the nucleocytoplasmic shuttling of KRIT1 depends critically on the integrity of its C-terminal PTB subdomain and is modulated by its N-terminal 207-amino-acid arm (aa 1–207), suggesting a novel mechanism whereby a signal-regulated conformational switch from the closed to the open state dictates KRIT1 nucleocytoplasmic shuttling and intermolecular interactions, thus presumably impacting its functions (Francalanci et al., 2009). Nevertheless, the upstream regulatory proteins and signaling mechanisms that control the subcellular localization of KRIT1 remain unclear.

Herein, we investigated potential upstream regulation of KRIT1 nucleocytoplasmic shuttling by members of the protein kinase C (PKC) family of serine/threonine kinases, including PKC isoforms involved in the control of physiological and pathological responses to oxidative stress and inflammation (Cosentino-Gomes et al., 2012; Giorgi et al., 2010; Gopalakrishna and Jaken, 2000; Inoguchi et al., 2003; Scoditti et al., 2014; Steinberg, 2015; Yan et al., 2008). PKC enzymes are structurally defined by a highly conserved C-terminal catalytic domain and an N-terminal regulatory domain, which contains the binding sites for allosteric activators, including diacylglycerol (DAG) and tumor-promoting phorbol esters such as phorbol 12-myristate 13-acetate (PMA) (Mellor and Parker, 1998; Newton, 2001). Based on sequence homology, distinctive structural features in their N-terminal regulatory domain and mode of activation, PKCs can be subdivided into three subfamilies: classical (or conventional) PKCs (cPKCs; α, βI/βII and γ; encoded by *PRKCA*, *PRKCB* and *PRKCG*, respectively) are activated by DAG in a Ca^2+^-dependent manner; novel PKCs (nPKCs; δ, ɛ, η and θ; encoded by *PRKCD*, *PRKCE*, *PRKCH* and *PRKCQ*, respectively) are Ca^2+^-insensitive but are still activated by DAG; and atypical PKCs (aPKCs; ζ and ι/λ; encoded by *PRKCZ* and *PRKCI*, respectively) require neither DAG nor Ca^2+^ for their activation. Moreover, both cPKCs and nPKCs are targets of the tumor-promoting phorbol ester PMA, which activates these enzymes by eliminating the requirement for DAG, while aPKCs are insensitive to PMA (Mellor and Parker, 1998). Among their established functions, there is also evidence that PKCs are redox-sensitive kinases that phosphorylate Ser and Thr residues in many target proteins to regulate their molecular interactions and subcellular compartmentalization, including cytoplasmic and nuclear distribution and nucleocytoplasmic shuttling, in an isozyme-specific manner (Aisiku et al., 2011; Cosentino-Gomes et al., 2012; Doller et al., 2010; Giorgi et al., 2010; Goyal et al., 2005; Valovka et al., 2003).

Using distinct and complementary approaches, we found that PKC activation by PMA promotes cytoplasmic localization of KRIT1, whereas its inhibition leads to KRIT1 accumulation in the nucleus. Consistent with the redox-sensitive nature of both PKC and KRIT1 signaling, treatment with the antioxidant N-acetylcysteine was able to block PMA-induced translocation of KRIT1 from the nucleus to the cytoplasm. Furthermore, we showed that the PKC-dependent regulation of KRIT1 nucleocytoplasmic shuttling requires the integrity of its N-terminal domain, and is associated with phosphorylation of the Ser22 residue within this region. Lastly, we found that KRIT1 nucleocytoplasmic shuttling is mediated specifically by the PKCα isoform, suggesting that the activity of this enzyme may dictate distinct nuclear and cytoplasmic functions of KRIT1. Taken together, our results demonstrate for the first time a novel functional interaction between PKC signaling and KRIT1 subcellular dynamics, suggesting that a PKC-dependent modulation of KRIT1 nucleocytoplasmic shuttling may play an important role in redox signaling mechanisms implicated in cellular responses to oxidative stress.

## RESULTS

### PKC activation regulates the nucleocytoplasmic shuttling of KRIT1

**Fig. 1.**
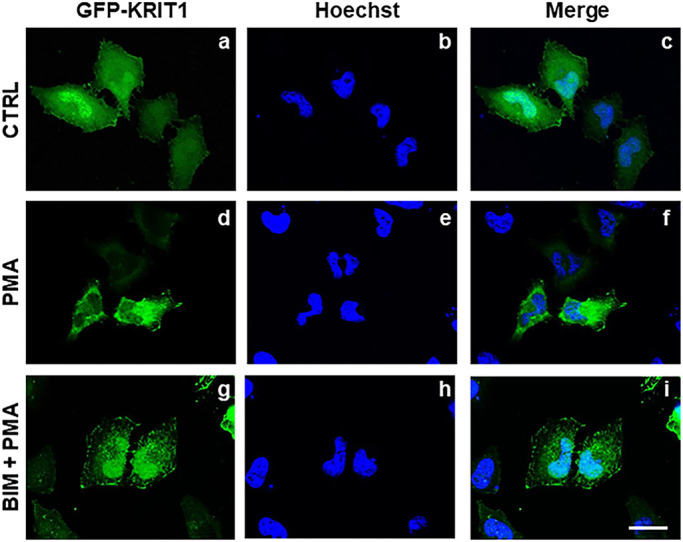
**PKC activity regulates the nucleocytoplasmic shuttling of KRIT1.** HeLa cells transiently transfected with a construct encoding GFP–KRIT1 were either (panels a–c) vehicle-treated (DMSO vehicle alone; CTRL), (panels d–f) treated with PMA (20 ng/ml for 2 h; PMA), or (panels g–i) pre-treated with BIM (1 µM for 30 min) before PMA treatment (BIM+PMA), and GFP–KRIT1 subcellular distribution was assessed by fluorescence microscopy. Nuclei were visualized with the DNA-specific blue fluorescent dye Hoechst. Images are representative of *n*>3 independent experiments. Notice that, as compared to the prevalent nuclear localization of GFP–KRIT1 in vehicle-treated cells (panels a–c), cell treatment with the PKC activator PMA resulted in a drastic shift in GFP–KRIT1 subcellular distribution towards an almost exclusively cytoplasmic localization (panels d–f), which was prevented by cell pre-treatment with the PKC inhibitor BIM (panels g–i), suggesting a role for PKC activation in regulation of KRIT1 nucleocytoplasmic shuttling. Scale bar: 15 μm.

To assess the potential role of PKC in regulating KRIT1 nucleocytoplasmic shuttling, non-confluent HeLa cells transiently transfected with an EGFP-tagged KRIT1 cDNA construct (GFP–KRIT1) (Francalanci et al., 2009) were treated with either vehicle alone or PMA, a well-established activator of cPKCs and nPKCs (Castagna et al., 1982), and analyzed by fluorescence microscopy to assess GFP–KRIT1 subcellular distribution, as described in Materials and Methods. As compared to the prevalent nuclear localization of GFP–KRIT1 in vehicle-treated cells (Fig. 1 panels a–c), cell treatment with PMA resulted in a drastic shift in GFP–KRIT1 subcellular distribution towards an almost exclusively cytoplasmic localization (Fig. 1 panels d–f), suggesting that KRIT1 is responsive to PMA-induced PKC activation. To assess whether the observed effect was indeed due to PKC activation, cells were pre-treated with bisindolylmaleimide I (BIM), a PKC inhibitor that acts as a competitive inhibitor for the ATP-binding site of PKC and shows high selectivity for PKCα, β1, β2, γ, δ, and ε isozymes, before treatment with PMA. In contrast to cell treatment with PMA alone (Fig. 1 panels d–f), pre-treatment with BIM before PMA treatment (BIM+PMA) did not affect the nuclear localization of GFP–KRIT1 (Fig. 1 panels g–i), confirming a specific role for PKC activation in the regulation of KRIT1 nucleocytoplasmic shuttling.

**Fig. 2.**
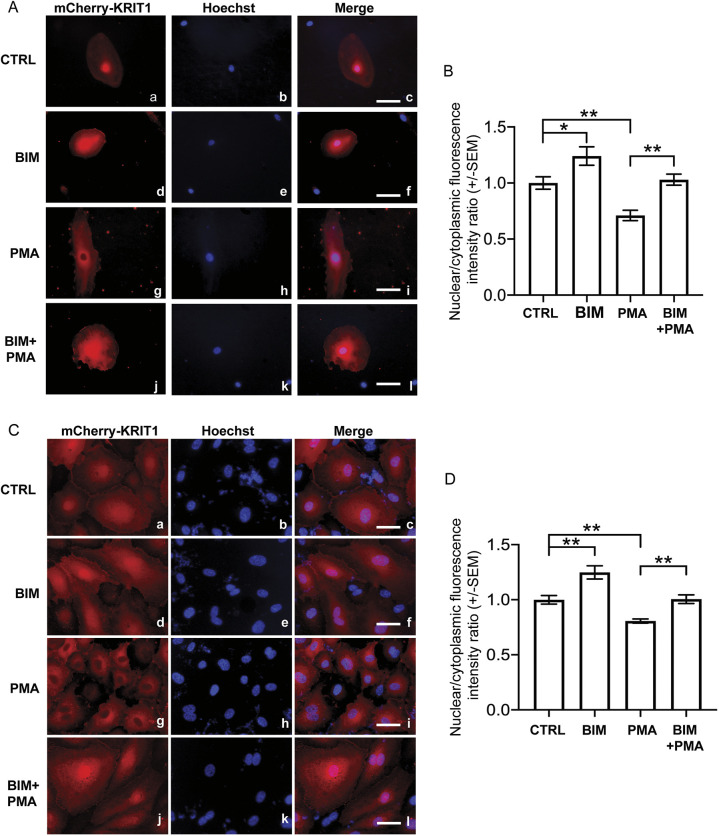
**The PKC-dependent nucleocytoplasmic translocation of KRIT1 occurs also in endothelial cells.** (A) Representative mCherry–KRIT1 fluorescence, nuclear staining (Hoechst) and merged images in adenovirally-transduced HPAECs. Subconfluent cells were treated with DMSO vehicle (CTRL; panels a–c), 1 µM BIM for 30 min (panels d–f), 20 ng/ml PMA for 2 h (panels g–i), or pre-treated for 30 min with the PKC-specific inhibitor BIM before PMA administration (BIM+PMA; panels j–l). Subcellular localization of mCherry–KRIT1 was analyzed by epifluorescence microscopy. Consistent with observations in HeLa cells, PMA promoted KRIT1 translocation from the nucleus to cytoplasm, while BIM treatment promoted nuclear accumulation. Scale bars: 20 μm. (B) Quantification of nuclear:cytoplasmic fluorescence intensity ratio. *n*=36 cells from five biological replicates. (C) Representative mCherry–KRIT1 fluorescence, nuclear staining (Hoechst) and merged images in adenovirally-transduced HPAECs. Confluent cells were treated with DMSO vehicle (CTRL; panels a–c), 1 μM BIM for 30 min (panels d–f), 20 ng/ml PMA for 2 h (panels g–i), or pre-treated for 30 min with BIM prior to PMA treatment (BIM+PMA; panels j–l). Subcellular localization of mCherry–KRIT1 was analyzed by epifluorescence microscopy. PMA treatment strongly promoted nuclear-to-cytoplasmic shuttling of KRIT1 in confluent endothelial cells, while BIM treatment with or without PMA promoted KRIT1 nuclear accumulation, similar to effects seen in subconfluent cells. Scale bars: 50 μm. (D) Quantification of nuclear:cytoplasmic fluorescence intensity ratio. *n*=37 cells from three biological replicates. Data in B and D are mean±s.e.m. ratios normalized to CTRL, *P<0.05; **P<0.01 versus vehicle (one-way ANOVA with Tukey post hoc testing).

To investigate whether the PKC-dependent nucleocytoplasmic shuttling of KRIT1 observed in HeLa cells could be recapitulated in an endothelial cellular model, which is more closely related to CCM disease, we tested the effects of PMA and BIM+PMA treatments on primary human pulmonary artery endothelial cells (HPAECs) transiently transduced with adenoviral mCherry–KRIT1. To make an accurate comparison with the data obtained with HeLa cells, cells without cell‒cell contacts were analyzed. Consistent with our observations in HeLa cells, subconfluent HPAECs treated with PMA exhibited a significantly enhanced cytoplasmic localization of mCherry–KRIT1 (Fig. 2A, panels g–i) as compared to their vehicle-treated counterparts (Fig. 2A, panels a–c), whereas mCherry–KRIT1 nuclear localization was not affected in HPAECs treated with either BIM alone (Fig. 2A, panels d–f) or BIM+PMA (Fig. 2A, panels j–l), suggesting that PKC activation induces KRIT1 nucleocytoplasmic shuttling in both epithelial and endothelial cells. The ratio of nuclear-to-cytoplasmic localization was quantified by comparison of fluorescence intensities (Fig. 2B). Remarkably, the PKCα/β selective inhibitor Gö6976 was as effective as BIM in preventing PMA-induced nucleus-to-cytoplasm translocation of KRIT1 in both HeLa and HPAECs (data not shown), suggesting a major role for these PKC isoforms.

Because endothelial cells grow as a monolayer *in vivo*, we next wanted to investigate whether the effect of PKC activation on KRIT1 localization occurred in the presence of intact endothelial cell–cell contacts. In confluent endothelial cells, KRIT1 has been reported to have a mixed localization, and is found in the nucleus and cytoplasm, as well as at sites of cell–cell contact in resting cells. When we treated a confluent monolayer of mCherry–KRIT1-expressing HPAECs with PMA, we saw a decrease in KRIT1 nuclear localization (Fig. 2C, panels g–i) as compared to that in their vehicle-treated counterparts (Fig. 2C, panels a–c), again consistent with what was observed in HeLa and subconfluent endothelial cells. mCherry–KRIT1 nuclear localization was not affected by treatment with BIM alone (Fig. 2C, panels d–f), and pre-treatment with BIM was able to block the translocation of KRIT1 out of the nucleus induced by PMA (Fig. 2C, panels j–l). The ratio of nuclear-to-cytoplasmic localization in all conditions was quantified by comparison of fluorescence intensities in 37 cells from three biological replicates (Fig. 2D). KRIT1 localization to cell–cell contacts was also decreased by PMA-treatment and reversed by pre-treatment with BIM, which is unsurprising given the known destabilizing effects of PMA and PKC activation on endothelial junctions (Kumar et al., 2009; Sandoval et al., 2001). While these results indicate that PMA-induced nucleocytoplasmic shuttling occurs in the presence of cell–cell contacts, they also suggest that it may be related to other KRIT1 functions, including a potential functional relationship with redox-dependent mechanisms involved in the regulation of endothelial permeability through the modulation of antioxidant responses, such as those we have previously described (Goitre et al., 2010, 2017; Marchi et al., 2015).

### Nucleocytoplasmic shuttling of KRIT1 is inhibited by the antioxidant N-acetylcysteine

Cell treatment with PMA is known to induce reactive oxygen species (ROS) production via PKC-dependent activation of NADPH oxidase (Nox) enzymes (Kuwabara et al., 2015). Consistently, a growing body of research has shown that PKC isoforms are upstream regulators of Nox enzymes in various cell types, including phagocytes, vascular smooth muscle cells and endothelial cells (Cosentino-Gomes et al., 2012; Inoguchi et al., 2000; Kuwabara et al., 2015). In turn, the upregulation of ROS levels by PKC-dependent activation of Nox enzymes may amplify PKC signaling (Giorgi et al., 2010; Gopalakrishna and Jaken, 2000), and has been suggested to be involved in various pathophysiological conditions, including human cardiovascular diseases (Cosentino-Gomes et al., 2012).

**Fig. 3.**
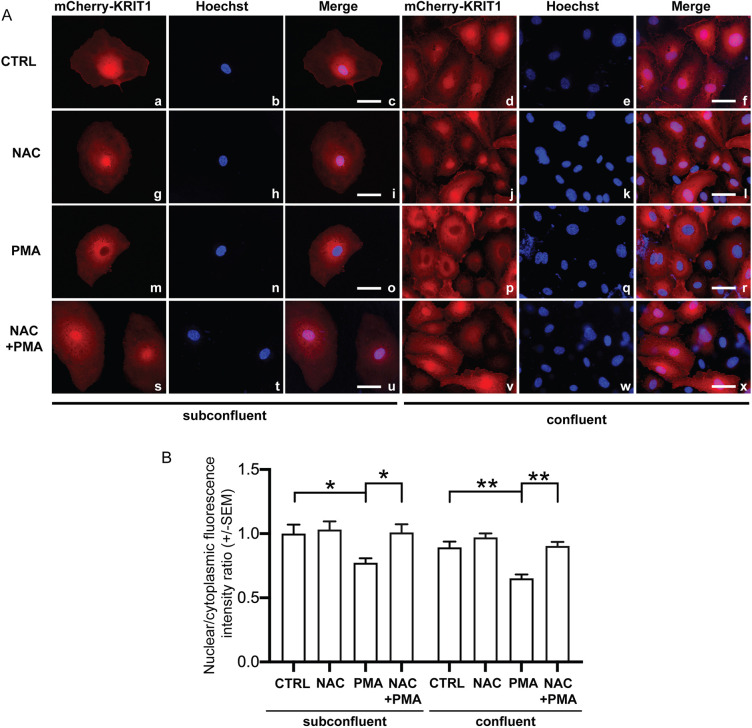
**NAC treatment promotes nuclear accumulation of KRIT1 in PMA-treated endothelial cells.** (A) Representative mCherry–KRIT1 fluorescence, nuclear staining (Hoechst), and merged images in adenovirally-transduced HPAECs. Subconfluent and confluent endothelial cells were treated with DMSO vehicle (CTRL; panels a–f), 20 ng/ml PMA for 2 h (panels g–l), 10 mM NAC for 2 h (panels m–r), or co-treated with 10 mM NAC and 20 ng/ml PMA for 2 h (NAC+PMA; panels s–x). Subcellular localization of mCherry–KRIT1 was analyzed by epifluorescence microscopy. While PMA promoted KRIT1 translocation out of the nucleus, NAC treatment alone or in conjunction with PMA treatment promoted nuclear localization of KRIT1, indicating a role for redox-mediated control of PKC activation in KRIT1 nucleocytoplasmic shuttling. Scale bars: 20 µm (subconfluent), 50 μm (confluent). (B) Quantification of nuclear:cytoplasmic fluorescence intensity ratio. Data shown are mean±s.e.m. ratios normalized to CTRL. Subconfluent, *n*=18 cells from four biological replicates. Confluent, *n*=28 cells from three biological replicates. *P<0.05; **P<0.01 versus control (one-way ANOVA with Tukey post hoc testing).

We have previously shown that KRIT1 is involved in redox homeostasis and signaling, including the regulation of NADPH oxidase-dependent ROS production (Goitre et al., 2010, 2014, 2017), suggesting that the PKC-dependent regulation of KRIT1 subcellular localization could be redox sensitive. To test this hypothesis, we treated subconfluent and confluent HPAECs with N-acetylcysteine (NAC), a commonly used antioxidant, in the presence and absence of PMA. Notably, the presence of NAC prevented KRIT1 nuclear-to-cytoplasmic translocation induced by PMA (Fig. 3), demonstrating that the PKC-dependent nucleocytoplasmic translocation of KRIT1 is indeed a redox-dependent mechanism, and raising the possibility that it represents an antioxidant defense response to pro-oxidant conditions known to be induced upon PKC activation (Giorgi et al., 2010; Gopalakrishna and Jaken, 2000; Joo et al., 2015; Kumar et al., 2009). Studies are now ongoing to further address this possibility.

### The N-terminal region is crucial for KRIT1 nucleocytoplasmic shuttling upon PKC activation

Previously, we identified KRIT1B, a KRIT1 isoform characterized by the alternative splicing of the fifteenth coding exon, which causes the deletion of a 39-amino-acid segment (aa 676–714) forming the distal β-sheet of the F3/PTB-like subdomain of the FERM domain (Retta et al., 2004). This isoform exhibits an exclusive cytoplasmic localization despite the presence of a functional nuclear localization sequence (NLS) at the N-terminus of the protein (_46_KKKRKK_51_), suggesting that the C-terminal PTB-like subdomain enables the nucleocytoplasmic shuttling of KRIT1, while its alteration confers a restricted cytoplasmic localization (Francalanci et al., 2009). Indeed, by taking advantage of the KRIT1B isoform and performing site-directed mutagenesis, we could demonstrate that an intact FERM domain (aa 419–736) is necessary and sufficient for KRIT1 nuclear translocation, whereas the KRIT1 N-terminal region acts mainly as a regulatory arm that counterbalances the nuclear translocation property of the C-terminal region (Francalanci et al., 2009). Consistently, a KRIT1 deletion mutant lacking the N-terminal arm (207 amino acids) shows constitutive and exclusive localization in the nucleus (Francalanci et al., 2009).

**Fig. 4.**
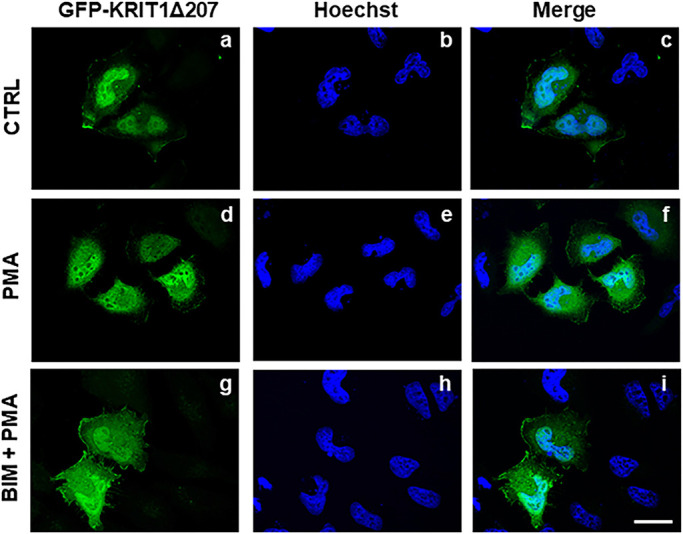
**The N-terminal domain plays a crucial role in KRIT1 nucleus- to-cytoplasm translocation induced by PKC activation.** HeLa cells transiently transfected with a construct encoding GFP–KRIT1Δ207, a GFP-tagged KRIT1 deletion mutant lacking the N-terminal domain (207 amino acids), were treated with (panels a–c) DMSO vehicle (CTRL), (panels d–f) PMA or (panels g–i) pre-treated with BIM before PMA administration (BIM+PMA), and the subcellular distribution of GFP–KRIT1Δ207 was assessed by fluorescence microscopy. Nuclei were visualized with the DNA-specific blue fluorescent dye Hoechst. Images are representative of *n*>3 independent experiments. Notice that the absence of the N-terminal region impaired KRIT1 impaired ability to translocate from the nucleus to cytoplasm upon PKC activation. Scale bar: 15 μm.

To evaluate the role of this N-terminal arm in the observed PKC-mediated nucleocytoplasmic shuttling of KRIT1, we transiently transfected HeLa cells with a construct encoding a GFP-tagged KRIT1 deletion mutant devoid of the N-terminal 207 amino acids (GFP–KRIT1Δ207) and performed fluorescence microscopy analysis to assess its subcellular localization in response to PMA-induced PKC activation. In agreement with previous results (Francalanci et al., 2009), the expression of the N-terminal deletion mutant GFP–KRIT1Δ207 in HeLa cells resulted in its constitutive nuclear accumulation (Fig. 4 panels a–c). However, in contrast to full length KRIT1 (Fig. 1 panels d–f), this N-terminal truncated mutant showed only very little, if any, change in its nuclear localization upon cell treatment with PMA (Fig. 4 panels d–f), with a consequently almost undetectable effect of cell pre-treatment with BIM (Fig. 4 panels g–i). While this evidence does not exclude that KRIT1 C-terminal domains may be partially responsive to PKC activation, it clearly demonstrates that the N-terminal regulatory domain plays a major role in PKC-dependent nucleocytoplasmic shuttling of KRIT1.

### KRIT1 is phosphorylated at Ser/Thr residues upon PKC activation

**Fig. 5.**
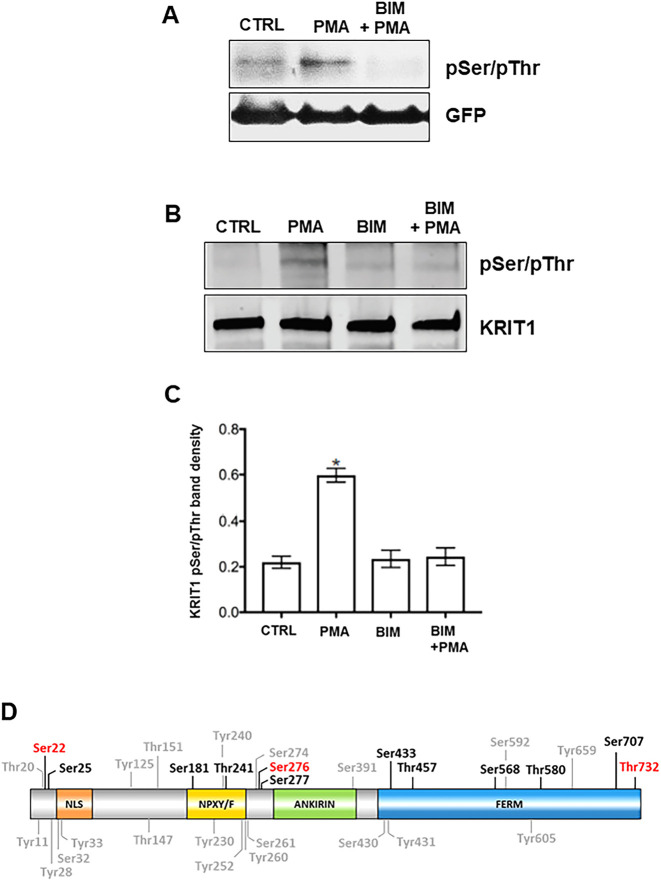
**PMA treatment induces Ser/Thr phosphorylation of KRIT1.** (A) HeLa cells transiently transfected with GFP–KRIT1 were treated with vehicle (CTRL), PMA or PMA+BIM. Whole-cell extracts were subjected to immunoprecipitation using anti-GFP antibody, and Ser/Thr phosphorylation of KRIT1 was detected with antibodies against pSer/Thr by western blotting analysis. Blots were probed for GFP–KRIT1 as a loading control for immunoprecipitation. Notice that after PMA treatment, a marked phosphorylation of KRIT1 in Ser/Thr residues was detectable. BIM pre-treatment completely prevented PMA-induced KRIT1 phosphorylation. Blot shown is representative of three experiments. (B) Representative pSer/pThr blot of transduced HPAEC lysates after immunoprecipitation with a specific anti-KRIT1 antibody. Blots were probed for total KRIT1 as loading control for immunoprecipitation. Bands shown (100 kDa) are mCherry–KRIT1; endogenous KRIT1 expression is below the antibody detection limit. Consistent with results from HeLa cells, PMA treatment induced KRIT1 Ser/Thr phosphorylation. (C) Densitometry analysis of pSer/pThr in HPAEC. Data shown are mean±s.e.m. band density. *n*=6, *P<0.05 versus vehicle (one-way ANOVA with Tukey post hoc testing). (D) General KRIT1 phosphorylation sites reported in available phosphorylation databases (Ser, Thr and Tyr residues indicated in light gray), and potential PKC-specific KRIT1 Ser and Thr phosphorylation sites predicted by our analysis with GPS 5.0 (Ser and Thr residues indicated in black and red colors, where the red color serves to highlight residues also reported in phosphorylation databases).

It has been established that individual PKC isoforms regulate subcellular compartmentalization and nucleocytoplasmic shuttling of target proteins by triggering the simultaneous phosphorylation of different phosphorylation sites within their regulatory domains (Aisiku et al., 2011; Doller et al., 2010; Goyal et al., 2005; Valovka et al., 2003). In this light, we sought to determine whether the observed nucleus-to-cytoplasm translocation of KRIT1 in response to PKC activation by PMA could be associated with any PKC-mediated phosphorylation of KRIT1. To this end, HeLa cells transiently transfected with EGFP-tagged KRIT1 (GFP–KRIT1) (Francalanci et al., 2009) and mCherry–KRIT1-expressing HPAECs were treated with PMA, BIM, BIM+PMA or vehicle (CTRL), and then lysed for subsequent immunoprecipitation and western blotting analysis of Ser/Thr phosphorylation, as described in Materials and Methods. Specifically, to assess potential changes in Ser/Thr phosphorylation levels, GFP–KRIT1 and mCherry–KRIT1 were immunoprecipitated from cell lysates with anti-GFP and anti-KRIT1 antibodies, respectively, and analyzed by western blotting with pan-phospho-Ser/Thr antibodies. As compared to the almost undetectable Ser/Thr phosphorylation of both GFP–KRIT1 and mCherry–KRIT1 in vehicle-treated HeLa cells (CTRL; Fig. 5A) and HPAECs (CTRL; Fig. 5B,C), respectively, treatment of these cell types with PMA resulted in a significant increase in Ser/Thr phosphorylation levels (PMA; Fig. 5A–C). Ser/Thr phosphorylation was rescued by cell pre-treatment with the PKC inhibitor BIM (BIM+PMA; Fig. 5A–C), suggesting that KRIT1 undergoes Ser/Thr phosphorylation in response to PMA-induced PKC activation in both epithelial and endothelial cells.

Consistent with our findings, various high-throughput proteomic studies have indeed demonstrated that KRIT1 can undergo phosphorylation at multiple sites, including Tyr11 (1), Thr20 (2), Ser22 (29), Tyr28 (1), Ser32 (3), Tyr33 (1), Tyr125 (1), Thr147 (1), Thr151 (14), Tyr230 (3), Tyr240 (1), Tyr252 (3), Tyr260 (8), Ser261 (2), Ser274 (7), Ser276 (13), Ser391 (1), Ser430 (1), Tyr431 (1), Ser592 (4), Tyr605 (1), Tyr659 (1) and Thr732 (1), where the number in parentheses indicates the number of studies referring to a specific phosphorylated amino acid residue, as resulting from available phosphorylation databases, including PhosphoSitePlus (Hornbeck et al., 2015), ProteomeScout (Matlock et al., 2015), PhosphoNet (Safaei et al., 2011), qPhos (Yu et al., 2019), and NetXProt (Duek et al., 2018). In order to predict PKC-specific phosphorylation sites, KRIT1 sequence was analyzed using the bioinformatic tool Group-based Prediction System (GPS) 5.0 (Wang et al., 2020; Zhou et al., 2004). Results of this analysis revealed several potential PKC-specific KRIT1 phosphorylation sites, including Ser22, Ser25 and Thr181 located within the N-terminal 207-amino-acid regulatory region of KRIT1 (Fig. 5D and Table 1).

**Table 1.**
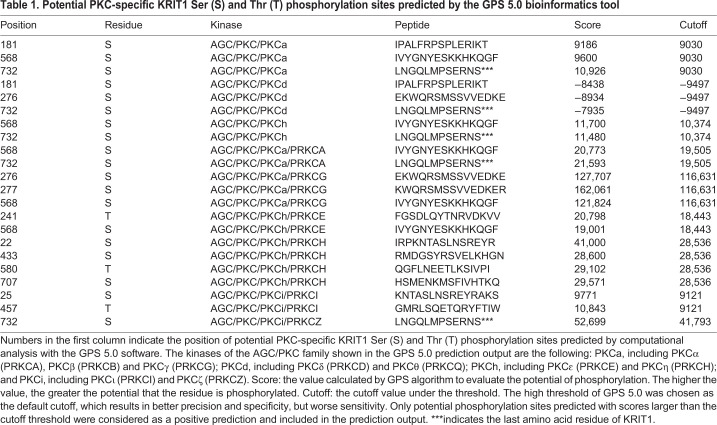
**Potential PKC-specific KRIT1 Ser (S) and Thr (T) phosphorylation sites predicted by the GPS 5.0 bioinformatics tool**

**Fig. 6.**
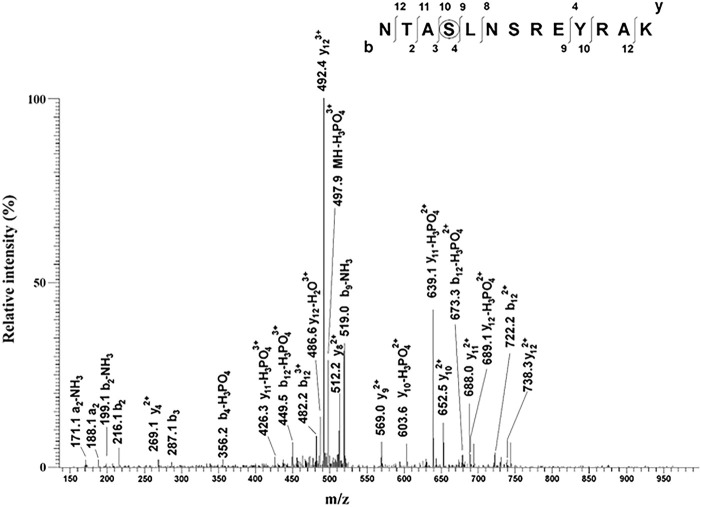
**Mass spectrometric characterization of the phosphopeptide (19–31)P identified in the endoproteinase LysC digest of GFP–KRIT1 from HeLa cells treated with PMA.** GFP–KRIT1 proteins from PMA-treated and control HeLa cells were immunoprecipitated with a specific anti-GFP antibody and separated by SDS–PAGE. GFP–KRIT1 bands were then excised, *in gel* digested with endoproteinase LysC and subjected to peptide mapping experiments using nLC-ESI-LIT-MS/MS. Shown is the fragmentation mass spectrum of the triply charged ion at *m/z* 530.8 associated with the peptide (19–31)P, which was uniquely observed in the GFP–KRIT1 sample from HeLa cells treated with PMA. Peptide numbering refers to the protein sequence lacking the GFP portion. Observed fragment ions assigned a phosphorylation site at Ser22.

To test whether any of these predicted PKC-specific KRIT1 phosphorylation sites were indeed phosphorylated upon PKC activation by cell treatment with PMA, we performed a dedicated phosphoproteomic analysis of GFP–KRIT1 immunoprecipitated from HeLa cells either vehicle-treated or treated with PMA. Specifically, GFP–KRIT1 was immunoprecipitated from lysates of both vehicle-treated and PMA-treated cells using a highly specific GFP antibody, and immunocomplexes were separated by SDS–PAGE. Protein bands corresponding to GFP–KRIT1 were then excised from the gel and digested in parallel with the lysyl endoproteinase LysC. The resulting protein digests were then subjected to nLC–ESI–LIT–MS/MS (nano-liquid chromatography-electrospray ionization-linear ion trap-tandem mass spectrometry) analysis. As shown in Fig. 6, only the phosphopeptide (19–31)P was observed in the endoproteinase LysC digest of KRIT1 from PMA-treated cells. Its fragmentation spectrum unequivocally assigned phosphorylation at Ser22. This phosphorylated component was absent in the digest of KRIT1 from control vehicle-treated cells, which uniquely displayed the non-modified counterpart (peptide 19–31). No additional information on phosphorylation sites was obtained when GFP–KRIT1 from transfected HeLa cells treated with PMA or vehicle was digested with endoproteinase AspN (data not shown). While confirming previous investigations pointing to Ser22 as a KRIT1 phosphorylation site of high-stoichiometry, our results proved that KRIT1 phosphorylation at this site may occur as a consequence of PKC activation, suggesting a potential regulatory role in the observed PKC-dependent nucleocytoplasmic shuttling of KRIT1.

To determine the contribution that the Ser22 residue has in the nucleocytoplasmic shuttling of KRIT1, we replaced Ser22 with alanine (S22A) using a previously described KRIT1 cDNA mutagenesis and cloning procedure (Francalanci et al., 2009). The resulting EGFP-tagged KRIT1 S22A expression construct was transiently transfected into HeLa cells, and the subcellular distribution of GFP–KRIT1 S22A mutant protein was analyzed by fluorescence microscopy. The experimental outcomes showed that the S22A substitution did not significantly affect the predominant nuclear localization of KRIT1 in basal conditions or its translocation from the nucleus to the cytoplasm upon cell treatment with PMA (Fig. S1), suggesting that the Ser22 residue alone is not sufficient to regulate the PKC-dependent nucleocytoplasmic shuttling of KRIT1. Indeed, consistent with the potential involvement of additional KRIT1 phosphorylation sites of lower stoichiometry, there is evidence that PKC activity-dependent regulation of nucleocytoplasmic shuttling of target proteins may require simultaneous phosphorylation of different phosphorylation sites (Doller et al., 2010). Further site-directed mutagenesis studies aimed at addressing the effects of either single or combined mutations of distinct KRIT1 phosphorylation sites are underway to address this possibility.

### PKCα is the major PKC isoform that regulates KRIT1 nucleus-to-cytoplasm translocation

Pharmacological modulators of PKC activity, including PMA and BIM, allowed us to demonstrate a major role for PKC in the control of KRIT1 nucleocytoplasmic shuttling. Nonetheless, given that both PMA and BIM compounds act on multiple PKC isoforms, as well as that distinct PKC isoforms have been implicated in nucleocytoplasmic shuttling of various proteins, it remained unclear whether one or more PKC isoforms were required. Based on the outcomes of *in silico* prediction of PKC-specific KRIT1 phosphorylation sites using the GPS 5.0 bioinformatics tool, PKCα and PKCδ isoforms emerged as major candidate regulators of KRIT1 nucleocytoplasmic shuttling.

**Fig. 7.**
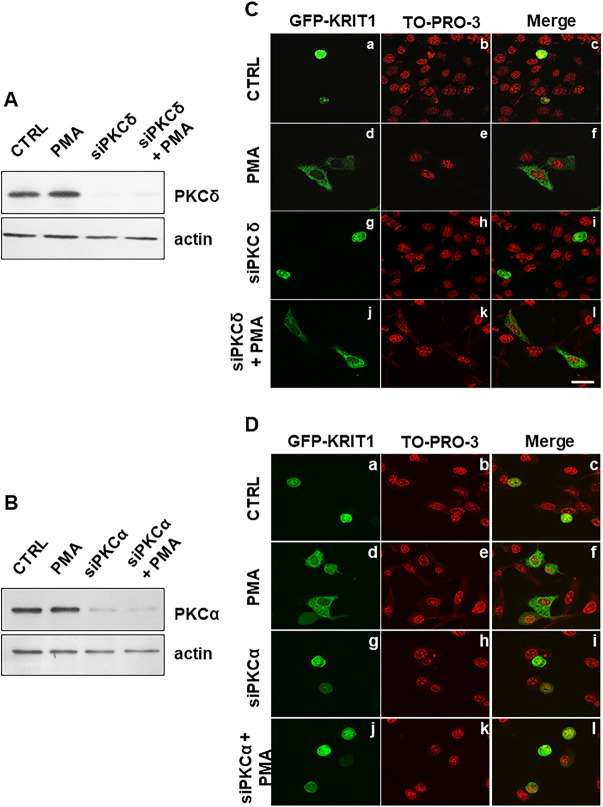
**PKCα is the major PKC isoform that regulates KRIT1 nucleus-to-cytoplasm translocation.** (A–D) HeLa cells transiently co-transfected with a construct encoding GFP–KRIT1 and either PKCδ siRNA, PKCα siRNA or scrambled siRNA, were either vehicle-treated (CTRL) or treated with PMA (20 ng/mL for 2 h; PMA), and analyzed by (A,B) western blotting and (C,D) fluorescence microscopy to assess the effectiveness of (A) PKCδ and (B) PKCα silencing and the relative subcellular distribution of GFP–KRIT1, respectively. (A,B) Efficiency of siRNA-mediated PKCδ (A; siPKCδ) and PKCα (B; siPKCα) silencing, as assessed by western blotting analysis with antibodies specific for either PKCδ or PKCα isoforms. β-actin was used as loading control. Notice that, whereas siRNA-mediated silencing resulted in a complete knockdown of either PKCδ or PKCα, PMA treatment had no effect on their expression. (C,D) Confocal fluorescence microscopy analysis of the effects of PMA treatment on GFP–KRIT1 subcellular localization in HeLa cells before (panels a–f) and after silencing of either PKCδ (C, panels g–l) or PKCα (D, panels g–l). Nuclei were visualized with the red fluorescent dye TO-PRO-3. Images are representative of three independent experiments. Notice that the silencing of PKCδ did not prevent the capacity of PMA treatment to induce KRIT1 nucleocytoplasmic translocation, suggesting that this PKC isoform does not play a role in the regulation of KRIT1 nucleocytoplasmic shuttling. In contrast, the silencing of PKCα prevented the capacity of PMA treatment to induce KRIT1 nucleocytoplasmic translocation, suggesting that this PKC isoform indeed plays a major role in the regulation of KRIT1 nucleocytoplasmic shuttling. Scale bar: 15 μm.

In order to validate these predictions, we used an RNA-interference approach to individually silence PKCα and PKCδ isoforms in HeLa cells expressing GFP–KRIT1. Western blotting analysis confirmed the effective knockdown of both PKCδ (Fig. 7A) and PKCα (Fig. 7B) isoforms, as well as that cell treatment with PMA did not affect the expression of either isoform (Fig. 7A,B). As clearly shown by confocal fluorescence microscopy analyses (Fig. 7C,D), the knockdown of PKCδ was ineffective in preventing KRIT1 nucleus-to-cytoplasm translocation induced by cell treatment with PMA (Fig. 7C), whereas the knockdown of PKCα was unequivocally effective (Fig. 7D), suggesting that PKCα plays a major role in the regulation of KRIT1 nucleocytoplasmic shuttling.

## DISCUSSION

Since its original discovery as a novel interactor of the small GTPase Rap1 (Serebriiskii et al., 1997), as well as being the major protein involved in the pathogenesis of the human genetic disease CCM (Laberge-le Couteulx et al., 1999; Sahoo et al., 1999), KRIT1 has progressively emerged as a key player in fundamental cellular functions, including control of cell–matrix and cell–cell adhesion (Glading et al., 2007; Zhang et al., 2001), Rho GTPase activity and actin cytoskeleton dynamics (Stockton et al., 2010), intracellular redox homeostasis and signaling (Antognelli et al., 2018a,b; Cianfruglia et al., 2019; Goitre et al., 2010, 2014), and autophagy (Marchi et al., 2015). The pleiotropic functions of KRIT1 have been clearly implicated in the maintenance of endothelial cell homeostasis and blood-brain barrier integrity through the control of coordinated molecular and cellular responses to oxidative stress and inflammation, which in turn suggest novel mechanisms of CCM disease onset and severity, providing new preventive and therapeutic perspectives (Antognelli et al., 2020; Choquet et al., 2016; De Luca et al., 2018; Finetti et al., 2020; Gibson et al., 2015; Goitre et al., 2017; Kim et al., 2020; Marchi et al., 2016; Perrelli et al., 2018; Retta and Glading, 2016; Trapani and Retta, 2015). On the other hand, recent evidence demonstrates that the consequences of KRIT1 loss-of-function mutations extend beyond the pathogenesis of CCM disease, being also implicated in the development of aortic endothelial dysfunction and atherosclerosis (Vieceli Dalla Sega et al., 2019), as well as of epithelial barrier dysfunction in the gastrointestinal tract (Wei et al., 2020). Consistent with its emerging functions in distinct tissues, KRIT1 has been shown to be expressed almost ubiquitously. Furthermore, it is also well established that KRIT1 can reside both in the cytoplasm and in the nucleus, implying that at least some of its diverse functions may be related to its nuclear localization (Draheim et al., 2017; Francalanci et al., 2009; Su et al., 2020; Zawistowski et al., 2005; Zhang et al., 2007). Though the nuclear functions of KRIT1 are still rather mysterious (Marzo et al., 2014), the identification of upstream regulatory proteins and signaling mechanisms that dictate KRIT1 nucleocytoplasmic shuttling may provide useful insights into the understanding of such functions.

In this study, we demonstrated for the first time that PKC plays a key role in regulating KRIT1 shuttling between the nucleus and the cytoplasm in epithelial and endothelial cells. In particular, using fluorescently-labeled KRIT1 constructs, we observed nuclear-to-cytoplasmic translocation of KRIT1 in response to PMA-mediated PKC activation (Figs 1, 2), which was prevented by PKC inhibitors, including BIM (Figs 1, 2) and Gö6976 (data not shown), as well as by antioxidant compounds, such as N-acetylcysteine (NAC) (Fig. 3). The PKC-dependent and redox-sensitive nucleocytoplasmic translocation of KRIT1 was detected in both subconfluent and confluent endothelial cells (Figs 2, 3), suggesting that it occurs independently of changes in cell density, thus pointing to a novel mechanism whereby KRIT1 subcellular localization is regulated through a redox-sensitive PKC signaling. Specifically, RNA-interference studies identified PKCα as the primary upstream regulator (Fig. 7). Consistently, there is clear evidence for a critical role of PKCα in the control of protein shuttling between the nucleus and the cytoplasm (Doller et al., 2007). While providing original insights into the regulation of KRIT1 subcellular dynamics, these findings raise also potential functional implications, including the intriguing possibility that the PKC-dependent nucleocytoplasmic shuttling of KRIT1 is a redox-sensitive mechanism implicated in cellular responses to oxidative stress.

Consistent with this hypothesis, a link between PKC functions and oxidative stress has been clearly established (Cosentino-Gomes et al., 2012; Giorgi et al., 2010; Gopalakrishna and Jaken, 2000; Joo et al., 2015; Kumar et al., 2009). In particular, PKC activation by PMA is known to promote ROS production, actomyosin contractility and adherens junction destabilization, leading to increased paracellular permeability (Gopalakrishna and Jaken, 2000; Joo et al., 2015; Kumar et al., 2009). Considering that we have previously shown that KRIT1 plays a major role in cellular defenses against oxidative stress and inflammation (Antognelli et al., 2020, 2018a,b; Goitre et al., 2017; Retta and Glading, 2016), including activation of the FoxO1–SOD2 axis (Goitre et al., 2010), inhibition of NADPH oxidases (Goitre et al., 2017) and stimulation of autophagy (Marchi et al., 2015), our current findings suggest that KRIT1 nucleocytoplasmic shuttling may counteract the pro-oxidant, destabilizing effects of PKC activation. This opens novel research avenues for a comprehensive characterization of the identified relationship between PKC and KRIT1. Among other ideas, it would be interesting to address whether the established capacity of PKCα to translocate from the cytosol into the nucleus upon various stimuli, including phorbol esters and redox changes (Giorgi et al., 2010; Schmalz et al., 1996), is somehow related to its ability to regulate KRIT1 nucleocytoplasmic shuttling. Furthermore, in light of the novel implication of PKC signaling in the regulation of KRIT1 subcellular trafficking, it would be interesting to assess whether and how it affects the interaction of KRIT1 with known binding partners, such as ICAP1α, Rap1 and CCM2, which were previously reported to be influenced by its distinct subcellular localizations (Draheim et al., 2017; Francalanci et al., 2009; Glading et al., 2007; Liu et al., 2011; Zawistowski et al., 2005). Though dedicated studies are needed to address the issue of whether antioxidant mechanisms and/or binding interactions are indeed influenced by KRIT1 nucleocytoplasmic shuttling, our findings provide critical support for this promising new research avenue.

Our discovery that the subcellular distribution of KRIT1 is regulated by PKC is corroborated by several studies showing that PKC plays a major role in regulating subcellular localization of a diverse variety of proteins, often as the result of direct phosphorylation (Andreeva et al., 2001; Doller et al., 2007; Represa et al., 1990; Topham et al., 1998; van Balkom et al., 2002). In this light, our demonstration that KRIT1 is a target for PKC-dependent phosphorylation events suggests a potential mechanism for PKC-dependent regulation of KRIT1 nucleocytoplasmic shuttling. Specifically, phospho-specific western blotting analysis showed that PKC activation induces Ser/Thr phosphorylation of KRIT1 (Fig. 5) and *in silico* predictive analysis of the KRIT1 amino acid sequence identified several residues that may be phosphorylated by PKC isozymes (Table 1 and Fig. 5D), including some in the KRIT1 N-terminal region. Consistently, a dedicated phosphoproteomic analysis showed that PKC activation results in the specific phosphorylation of a KRIT1 N-terminal serine residue (Ser22) (Fig. 6). However, mutation of Ser22 to a non-phosphorylatable alanine residue was unable to block the PMA-induced cytoplasmic translocation of KRIT1 (Fig. S1), suggesting the potential involvement of additional KRIT1 phosphorylation sites of lower stoichiometry. This agrees with previous reports that PKC activity-dependent regulation of nucleocytoplasmic shuttling of target proteins may require simultaneous phosphorylation of different phosphorylation sites (Doller et al., 2010). Dedicated studies based on site-directed mutagenesis are necessary to assign the specific contribution of each phosphorylation site present in KRIT1 with respect to protein nucleocytoplasmic translocation. Indeed, given the existence of multiple phosphorylation sites in KRIT1 (Fig. 5D), and the evidence that more than one phosphorylation event may be required for PKC-mediated regulation of protein nucleocytoplasmic shuttling (Doller et al., 2010), it is likely that distinct PKC-dependent phosphorylation sites contribute to a fine-tuned regulation of KRIT1 subcellular compartmentalization. Remarkably, the ability of both PKC and KRIT1 to reside in multiple subcellular compartments, including the nucleus, cytoplasm and plasma membrane, and serve a variety of cellular functions suggests that distinct functional relationships between PKC and KRIT1 may simultaneously occur in various subcellular locations, thus drawing a potential future avenue for research aimed at understanding how specific KRIT1 functions relate to where it resides within the cell. Further site-directed mutagenesis studies aimed at defining the effects of either single or combined mutations of distinct KRIT1 phosphorylation sites are underway to address this issue.

Herein, we have also provided evidence that the N-terminal 207-amino-acid region of KRIT1 is required for the ability of PKC to regulate KRIT1 nucleocytoplasmic shuttling, because a KRIT1 deletion mutant devoid of this region (KRIT1Δ207) was unresponsive to PKC activation, remaining confined to the nucleus (Fig. 4). This suggests that regulation of KRIT1 nucleocytoplasmic shuttling by PKC occurs in this region, which is consistent with our previous finding that the N-terminal region of KRIT1 is not required for KRIT1 translocation into the nucleus, but instead acts as a regulatory arm that counterbalances the constitutive nuclear translocation property of the C-terminal region (Francalanci et al., 2009).

The KRIT1 deletion mutant lacking the N-terminal 207-amino-acid region (KRIT1Δ207) (Francalanci et al., 2009) is devoid of both a functional NLS (aa 46–51) (Francalanci et al., 2009; Zawistowski et al., 2005) and the crucial binding site for ICAP1α (first NPXY motif, aa 192–195) (Zawistowski et al., 2002; Zhang et al., 2001), yet it has a constitutive nuclear localization and is unresponsive to PKC activation. Previous studies on the N-terminal region have revealed its role in ICAP1α binding (Béraud-Dufour et al., 2007), and suggested that this interaction promotes KRIT1 nuclear localization (Draheim et al., 2017; Francalanci et al., 2009; Su et al., 2020). Though our result may seem counterintuitive, one possible explanation is that the N-terminal region plays a major regulatory role in the release of the intramolecular autoinhibitory interaction between the F3/PTB lobe of the FERM domain at the C-terminus (aa 636–736) and the third NPXY motif at the N-terminus (aa 250–254), which may be required for KRIT1 nucleocytoplasmic shuttling (Francalanci et al., 2009). In this sense, loss of the N-terminal region may prevent conformational changes induced by PKC-dependent phosphorylation of KRIT1, thus disrupting nucleocytoplasmic shuttling. Indeed, there is evidence that the subcellular localization and functions of most FERM domain-containing proteins (FDCPs) are regulated by phosphorylation events that affect head-to-tail intramolecular autoinhibitory interactions involving the FERM domain, leading to the unmasking of important ligand-binding sites (Fehon et al., 2010; Frame et al., 2010; Goksoy et al., 2008; Goult et al., 2009; Li et al., 2007; Lietha et al., 2007; Mishra et al., 2012; Pearson et al., 2000). Furthermore, many FDCPs have been shown to reside both in the cytoplasm and in the nucleus, and to shuttle between these compartments in a FERM domain-dependent manner (Frame et al., 2010; Francalanci et al., 2009; Lim et al., 2012). Accordingly, all the FERM domains of these FDCPs have been shown to contain putative NES (nuclear export signal) and/or NLS (nuclear localization signal) sequences (Frame et al., 2010; Francalanci et al., 2009). On the other hand, it should be noted that the role of the KRIT1 N-terminal arm and C-terminal FERM domain may vary depending on the physiological context. For example, in confluent endothelial cells, a KRIT1 C-terminal construct lacking the first 204 residues has been shown to accumulate in cell–cell contacts, as well as in nuclei, whereas the KRIT1 N-terminus accumulates in the cytoplasm (Glading et al., 2007), suggesting that cellular context may elicit distinct combinations of regulatory signals, potentially including multiple phosphorylation events, which could have distinct functional consequences.

Taken together, these data point to PKCα as a novel regulator of KRIT1 subcellular localization, with potential implications in the regulation of the functions that KRIT1 is known to play in the biology of endothelial cells, including its established key role in redox signaling and antioxidant defenses. This insight may provide a new means for pharmacological regulation of KRIT1 localization, which would be beneficial in studying its functions within specific subcellular compartments, as well as in the development of novel targeted therapeutic strategies for CCM disease. Growing evidence demonstrates that KRIT1 can localize simultaneously in distinct subcellular compartments, including the nucleus, cytoplasm and plasma membrane, whereby it might participate in distinct signaling mechanisms to exert its emerging pleiotropic functions. Future work is required to distinguish the context-dependent cues that regulate KRIT1 localization and function in the distinct subcellular compartments. These cues will need to be unraveled in order to determine whether and how the nuclear versus cytoplasmic distribution of KRIT1 contributes to its known functions – including stabilization of endothelial and epithelial barriers, and cellular defenses against oxidative stress and inflammation – or regulates an as yet unknown function. Investigation of this question is precluded by our lack of understanding of the precise molecular mechanisms governing the nucleocytoplasmic shuttling of KRIT1. That is, despite the tantalizing observations that binding to various partners (i.e. ICAP1α) or phosphorylation by kinases (PKC) can affect the subcellular distribution of KRIT1, it is unclear how these upstream pathways control the physical translocation of KRIT1 through the nuclear pore, what role the NLS and NES sequences play, and the effect of conformational changes in the KRIT1 protein. Our work here presents an important first step in understanding this complex problem by pointing to PKC-dependent phosphorylation as a relevant mechanism.

## MATERIALS AND METHODS

### Cell culture

HeLa cells (ATCC, Manassas, VA, USA) were cultured in Dulbecco's modified Eagle's medium (DMEM; Gibco, Gaithersburg, MD, USA) supplemented with 10% Fetal Bovine Serum (FBS; Life Technologies, Carlsbad, CA, USA), 2 mM L-glutamine, 100 U ml^−1^ penicillin and 100 mg ml^−1^ streptomycin (EuroClone, Pero, MI, Italy).

Primary human pulmonary artery endothelial cells (HPAEC; Cell Applications Inc., San Diego, CA, USA) were cultured in DME/F-12 medium (HyClone GE Healthcare, Piscataway, NJ, USA) containing 5% FBS, 1× endothelial cell growth supplement (ECGS, ScienCell, Carlsbad, CA, USA), 15 U/ml heparin, 100 U/ml penicillin, 100 µg/ml streptomycin, and 0.25 µg/ml amphotericin B (Gibco). HEK293 cells (ATCC) were cultured for viral particle propagation in complete DMEM/high modified (Gibco) containing 10% FBS, 1× non-essential amino acids (Life Technologies), 100 U/ml penicillin, 100 µg/ml streptomycin, and 292 µg/ml L-glutamine. All cell lines were cultured at 37°C and 5% CO_2_ in a humidified incubator. All cell lines are authenticated upon receipt by PCR and tested after 15 passages (if not primary cells) for contamination.

### Plasmid constructs, siRNA, and transfections

Plasmid constructs encoding EGFP-tagged human KRIT1 (GFP–KRIT1) and EGFP-tagged KRIT1Δ207 (GFP–KRIT1Δ207) were cloned as previously described (Francalanci et al., 2009). A KRIT1 cDNA mutant carrying a serine-to-alanine mutation at codon 22 (S22A) was generated by oligodeoxyribonucleotide-directed site-specific mutagenesis (the AGT codon for Ser 22 was mutated to a GCT codon with the oligonucleotide 5′-ACTGCTGCTCTCAACTCCCGGGAG-3′). The mutated KRIT1 S22A cDNA was then cloned into the EcoRI–HindIII sites of pEGFP-C3 to generate a EGFP-tagged construct, as previously described (Francalanci et al., 2009). The resulting pEGFP–KRIT1 S22A expression construct was verified by sequencing.

HeLa cells were transfected with 5 µg of cDNA constructs using Fugene 6 Transfection Reagent (Roche), according to manufacturer's instructions. After an overnight incubation with the transfection mix, cells were washed, starved and subjected to treatments and analysis.

For PKC-silencing experiments, HeLa cells at 80% confluency were co-transfected with 4 μg of GFP–KRIT1 cDNA construct, 120 pmoles of isoform specific PKC siRNA (PKC alpha ON-TARGET plus SMART pool human PRKCA, and PKC delta ON-TARGET plus SMART pool human PRKCD; Dharmacon, Lafayette, CO, USA) and 10 μl of Lipofectamine 2000 (Thermo Fisher Scientific), according to manufacturer's instructions for siRNA and plasmid co-transfection. After 6 h of incubation with the transfection mix, the medium was replaced with fresh DMEM without antibiotics, and 24 h after transfection cells were subjected to treatments and analysis.

For KRIT1 expression in HPAEC, mCherry–KRIT1 was cloned into the adenoviral shuttle vector pDC315. The resulting plasmid (pDC315 mCherry–KRIT1) was co-transfected with the adenoviral parent plasmid pBHGloxΔE1,3Cre into HEK293 cells for viral propagation. Both viral vectors were gifts from Dr Alan Smrcka of the University of Michigan, Ann Arbor, MI, USA. Viral titer was measured using the immunoreactivity ‘spot’ assay (Duale et al., 2005). HPAEC (passage 2–5) at 50% confluence were transduced at a multiplicity of infection of 100; cells were subjected to treatments and analysis 24 h after transduction.

### Reagents and antibodies

Pharmacological compounds used for cell treatments were phorbol-12-myristate-13-acetate (PMA; Santa Cruz Biotechnology, Santa Cruz, CA; or Sigma-Aldrich, St Louis, MO, USA); PKC inhibitors, including bisindolylmaleimide-1 (BIM) and Gö6976 (Calbiochem, Bad Soden, Germany, or Cayman Chemical, Ann Arbor, MI, USA); and the ROS scavenger N-acetylcysteine (NAC; Sigma-Aldrich). Immunoprecipitation of GFP–KRIT1 and mCherry–KRIT1 was performed using rabbit polyclonal anti-GFP (ab290, 5 µg/1 mg cell protein; Abcam) and Mab15.B2 (5 µg/1 mg cell protein; Millipore, Burlington, MA) antibodies, respectively. Western blotting detection of KRIT1 was performed with either Mab15.B2 or our home-made rabbit polyclonal antibody against KRIT1 described previously (Goitre et al., 2010), whereas KRIT1 Ser/Thr phosphorylation was detected using specific anti-pan-phospho Ser/Thr antibodies (ab17464, 1:1000, Abcam; or 22A, 1:1000, BD Biosciences, San Jose, CA). PKCδ and PKCα were detected with specific rabbit polyclonal antibodies from Cell Signaling (#9374S, 1:1000) and Santa Cruz Biotechnology (sc-208, 1:5000), respectively. β-actin was detected with a mouse monoclonal antibody from Sigma-Aldrich (A5441, 1:1000). Primary antibodies were detected using affinity-purified HRP-conjugated anti-rabbit and anti-mouse secondary antibodies (from Sigma or GE Healthcare). Hoechst (H33258, Sigma) or TO-PRO-3 (Thermo Fisher Scientific, Waltham, MA, USA) were used for the staining of the nuclei. All antibodies were validated using antigen-negative conditions prior to use.

### Immunofluorescence and pharmacological treatments

HeLa cells were treated with 20 ng/ml PMA for 2 h, with or without a 30 min pre-treatment with 1 µM BIM. DMSO was used as vehicle control. After treatments, cells were fixed in 3% paraformaldehyde or cold methanol for 10 min, nuclei were stained with Hoechst or ToPro-3, and coverslips were mounted with Mowiol (Calbiochem) on microscope slides. Digital images were acquired with either an Axio-Observer-Z1 microscope (Zeiss) equipped with ApoTome system for optical sectioning or a three-channel TCS SP2 laser scanning confocal microscope (Leica Microsystems, Wetzlar, Germany).

HPAEC were plated on fibronectin-coated coverslips (10 μg/ml) and transduced in growth medium, then changed to serum-free DME/F-12 medium (HyClone, GE Healthcare, Piscataway, NJ) for 30 min. Cells were then treated with 20 ng/ml PMA for 2 h, with or without a 30 min pre-treatment with 1 µM BIM. DMSO was used as vehicle control. In addition, in experiments aimed at assessing whether the PKC-dependent regulation of KRIT1 localization was redox sensitive, cells were also treated with the ROS scavenger N-acetylcysteine (NAC; 10 mM) in the presence and absence of PMA. At the end of the treatment period, cells were fixed in 10% formalin and washed with 0.001% Triton X-100 in phosphate-buffered saline (PBS). Cells were counterstained with Hoechst 33258 (VWR, Radnor, PA) to label nuclei, then mounted on glass slides with ProLong Gold Antifade (Invitrogen, Carlsbad, CA). Images were acquired on an Olympus IX70 fluorescent microscope using a Hamamatsu digital imaging system. Fluorescence was quantified by calculating the ratio of pixel intensity in the nucleus to the average pixel intensity of four cytoplasmic regions halfway between the nucleus and cell edge.

Both the concentrations of pharmacological compounds and the time of treatment used were selected according to data found in the literature and the outcomes of our preliminary experiments. Fluorescence microscopy experiments were performed by distinct research groups and equipment from either Italy (Torino and Genova) or USA (Rochester, NY), which ensured reproducibility and specificity of the experimental outcomes.

### Immunoprecipitation and western blotting

GFP–KRIT1-transfected HeLa cells treated with PMA or BIM+PMA were lysed in NP-40 buffer (Sigma) containing protease and phosphatase inhibitors (P8340 and P2850, respectively; Sigma). GFP–KRIT1 was immunoprecipitated from cell lysates using the rabbit polyclonal anti-GFP antibody (ab290, Abcam), and analyzed by western blotting with pan-phospho-Ser/Thr antibody ab17464 (1:1000). Western blotting analysis was performed as previously described (Balzac et al., 2005).

mCherry–KRIT1-expressing HPAECs treated with PMA, BIM and BIM+PMA were lysed in buffer containing 20 mM HEPES-KOH pH 7.5, 1.5 mM MgCl_2_, 5 mM KCl, and protease and phosphatase inhibitors, supplemented with 1% Triton X-100. KRIT1 was immunoprecipitated (Mab15.B2, Millipore, Burlington, MA) from total lysate and blotted with pan-phospho-Ser/Thr antibody 22A (1:1000).

### *In silico* prediction of putative PKC-specific phosphorylation sites

Putative phosphorylation sites for the PKC family of the AGC (PKA, PKG, PKC) kinases were predicted using the bioinformatics tool Group-based Prediction System (GPS) 5.0 (http://gps.biocuckoo.org/) (Wang et al., 2020; Zhou et al., 2004). The high threshold of GPS 5.0 was chosen as the default cutoff, which results in better precision and specificity. Only potential phosphorylation sites predicted with scores larger than the cutoff threshold were considered as a positive prediction and included in the prediction output.

### Mass spectrometry analysis

GFP–KRIT1 protein immunocomplexes isolated by immunoprecipitation with an anti-GFP antibody from PMA-treated or control cell extracts containing protease and phosphatase inhibitors (P8340 and P2850, Sigma) were resolved by 10% SDS–PAGE. Bands corresponding to GFP–KRIT1 were then excised, *in-gel* alkylated with iodoacetamide, digested with endoproteinase LysC or endoproteinase AspN (Roche), and extracted as previously reported (Salzano et al., 2013). Peptide mixtures were directly subjected to peptide mapping experiments or further enriched for phosphopeptides by using Ga^3+^-immobilized metal ion affinity chromatography (Ga^3+^*-*IMAC; Phosphopeptide Isolation Kit, Pierce*,* USA) (D'Ambrosio et al., 2006).

All samples were analyzed by nLC-ESI-LIT-MS/MS using an LTQ XL mass spectrometer (ThermoFisher, San Jose, CA), equipped with a Proxeon nanospray source (Proxeon, Denmark) connected to an Easy-nanoLC (Proxeon). Peptide mixtures were separated on an Easy C_18_ column (10×0.075 mm, 3 µm) (Proxeon). Mobile phases consisted of 0.1% (v/v) aqueous formic acid (solvent A) and 0.1% (v/v) formic acid in acetonitrile (solvent B), running at flow rate of 300 nl/min. Solvent B ramped from 5% to 35% over 45 min, from 35% to 60% over 10 min, and from 60% to 95% over 20 min. Spectra were acquired in the range of *m/z* 400–2000. Acquisition was controlled by a data-dependent product ion scanning procedure over the three most abundant ions, enabling dynamic exclusion (repeat count of two and exclusion duration 60 s); the mass isolation window and collision energy values were set to *m/z* 3 and 35%, respectively. Raw data were searched by using Sequest (ThermoFisher) and Mascot (Matrix Science, UK) within the Proteome Discoverer software package version 1.0 SP1 (ThermoFisher) against an indexed database containing the GFP–KRIT1, LysC endoproteinase, AspN endoproteinase and common keratin sequences. Database searching was performed by selecting Cys carbamidomethylation as static and Met oxidation and Ser/Thr/Tyr phosphorylation as dynamic modifications. A mass tolerance value of 2 Da and 0.8 Da (for precursor ion and MS/MS fragments, respectively), endoproteinase LysC or endoproteinase AspN as proteolytic enzymes, and a missed cleavages maximum value of two were used as search parameters. Definitive assignment of peptide phosphorylation site(s) was associated with manual spectral visualization and verification.

### Statistical analysis

Data were analyzed using PRISM software (Version 7.0, GraphPad Software, La Jolla, CA). Unpaired one-way ANOVA was performed with Bonferroni or Tukey post hoc testing as indicated in figure legends.

## Supplementary Material

Supplementary information
