# Supplementary material for: Protein kinase Cα regulates the nucleocytoplasmic shuttling of KRIT1
Source: J Cell Sci. 2021 Feb 4;134(3):jcs250217. doi: 10.1242/jcs.250217 (PMC7875496; doi:10.1242/jcs.250217)
Supplement: Supplementary information [file joces-134-250217-s1.pdf]

# Figure S1

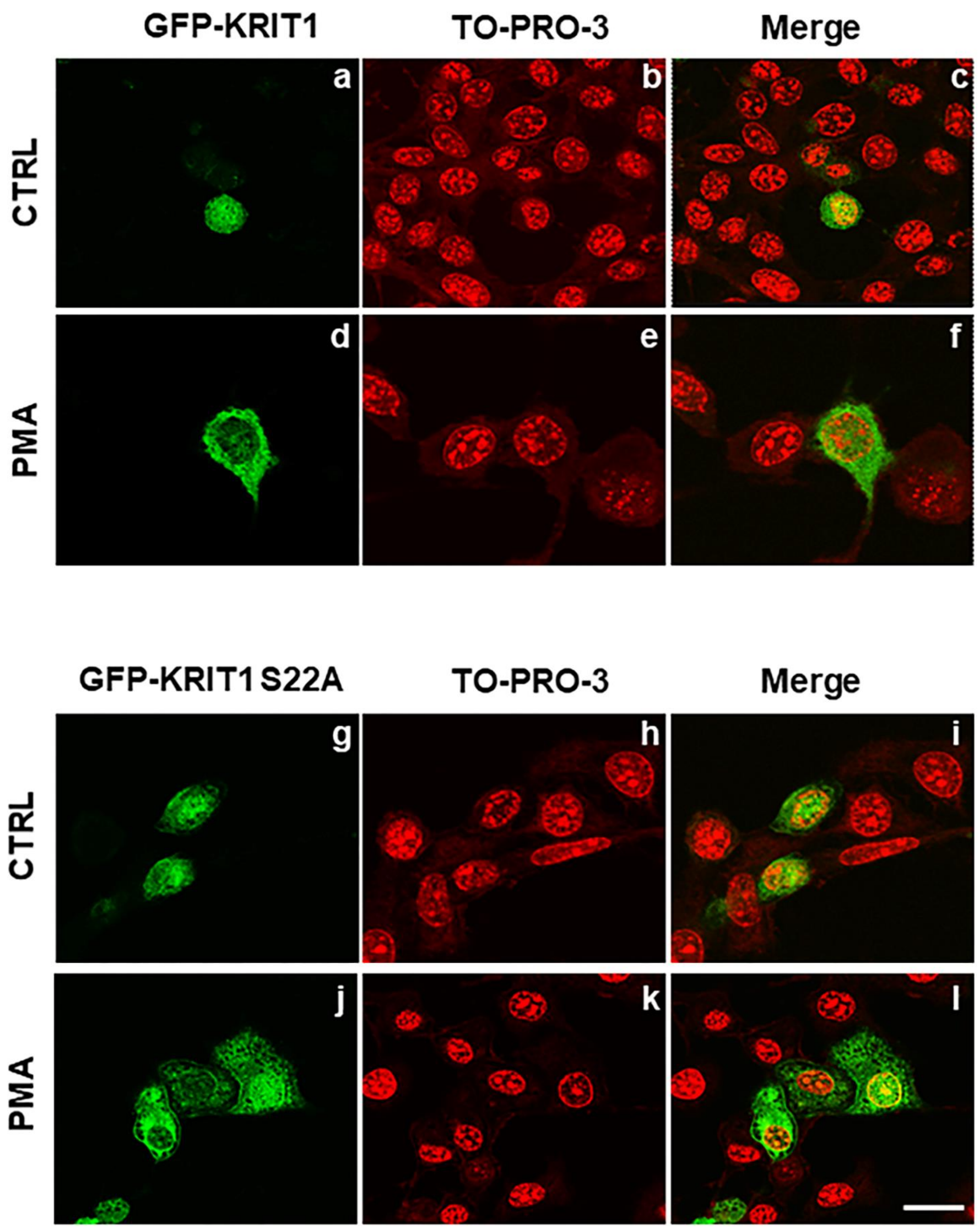


Figure S1. The S22A substitution does not affect the PKC-dependent nucleocytoplasmic shuttling of KRIT1.

HeLa cells transiently transfected with EGFP-tagged constructs encoding wildtype KRIT1 (GFP-KRIT1, panels a-f) or a KRIT1 mutant carrying the S22A substitution (GFP-KRIT1 S22A, panels g-l) were either vehicle-treated (DMSO vehicle alone) (CTRL, panels a-c and g-i) or treated with PMA (20 ng/mL for 2 h) (PMA, panels d-f and j-l), and analyzed by fluorescence microscopy. Nuclei were visualized with the red fluorescence dye TO-PRO-3. Images are representative of three independent experiments. Notice that both GFP-KRIT1 and GFP-KRIT1 S22A showed a prevalent nuclear localization in vehicle-treated cells (panels a-c and g-I, respectively), as well as a drastic shift towards an almost exclusively cytoplasmic localization upon cell treatment with the PKC activator PMA (panels d-f and j-l, respectively), suggesting that the S22A substitution does not affect the PKC-dependent nucleocytoplasmic shuttling of KRIT1. Scale bar represents 15 µm.

Journal of Cell Science • Supplementary information
